# Neutralization of extracellular histones by sodium-Β-O-methyl cellobioside sulfate in septic shock

**DOI:** 10.1186/s13054-023-04741-x

**Published:** 2023-11-24

**Authors:** Bruno Garcia, Fuhong Su, Laurence Dewachter, Yong Wang, Ning Li, Myriam Remmelink, Marie Van Eycken, Amina Khaldi, Raphaël Favory, Antoine Herpain, Anthony Moreau, Alexander Moiroux-Sahraoui, Francesca Manicone, Filippo Annoni, Lin Shi, Jean-Louis Vincent, Jacques Creteur, Fabio S. Taccone

**Affiliations:** 1 Experimental Laboratory of the Department of Intensive Care, Erasme Hospital, Université Libre de Bruxelles, 1070 Brussels, Belgium; 2 Department of Intensive Care, Centre Hospitalier Universitaire de Lille, Lille, France; 3 Laboratory of Physiology and Pharmacology, Université Libre de Bruxelles, Brussels, Belgium; 4 Grand Pharma (China) Co., Ltd, Wuhan, China; 5 Pathology Laboratory, Erasme Hospital, Hôpitaux Universitaires de Bruxelles, Université Libre de Bruxelles, Brussels, Belgium; 6 Department of Intensive Care, Erasme Hospital, Hôpital Universitaire de Bruxelles, Université Libre de Bruxelles, Brussels, Belgium

**Keywords:** Sepsis, Shock, Extracellular histones, Vasopressors, mCBS

## Abstract

**Background:**

Extracellular histones have been associated with severity and outcome in sepsis. The aim of the present study was to assess the effects of sodium-β-O-Methyl cellobioside sulfate (mCBS), a histone-neutralizing polyanion, on the severity and outcome of sepsis in an experimental model.

**Methods:**

This randomized placebo-controlled experimental study was performed in 24 mechanically ventilated female sheep. Sepsis was induced by fecal peritonitis. Animals were randomized to three groups: control, early treatment, and late treatment (*n* = 8 each). mCBS was given as a bolus (1 mg/kg) followed by a continuous infusion (1 mg/kg/h) just after sepsis induction in the early treatment group, and 4 h later in the late treatment group. Fluid administration and antimicrobial therapy were initiated 4 h T4 after feces injection, peritoneal lavage performed, and a norepinephrine infusion titrated to maintain mean arterial pressure (MAP) between 65–75 mmHg. The experiment was blinded and lasted maximum 24 h.

**Results:**

During the first 4 h, MAP remained > 65 mmHg in the early treatment group but decreased significantly in the others (*p* < 0.01 for interaction, median value at T4: (79 [70–90] mmHg for early treatment, 57 [70–90] mmHg for late treatment, and 55 [49–60] mmHg for the control group). mCBS-treated animals required significantly less norepinephrine to maintain MAP than controls (*p* < 0.01 for interaction) and had lower creatinine (*p* < 0.01), lactate (*p* < 0.01), and interleukin-6 (*p* < 0.01) levels, associated with reduced changes in H3.1 nucleosome levels (*p* = 0.02). Early treatment was associated with lower norepinephrine requirements than later treatment. Two control animals died; all the mCBS-treated animals survived.

**Conclusions:**

Neutralization of extracellular histones with mCBS was associated with reduced norepinephrine requirements, improved tissue perfusion, less renal dysfunction, and lower circulating IL-6 in experimental septic shock and may represent a new therapeutic approach to be tested in clinical trials.

**Supplementary Information:**

The online version contains supplementary material available at 10.1186/s13054-023-04741-x.

## Background

Sepsis is defined as “life-threatening organ dysfunction caused by dysregulated host responses to infection” [1]. Management of sepsis is based on early diagnosis, source control with antibiotic therapy, and hemodynamic support with fluids and vasopressors [2]. Sepsis remains a leading cause of admission to the intensive care unit (ICU) and of mortality worldwide, and new therapeutic strategies are needed [3].

Histones are positively charged nucleoproteins packaged with nuclear DNA into nucleosomes [4] and are released into the extracellular space after cell injury during cell death processes including necrosis, apoptosis, pyroptosis, and NETosis [5–7]. In sepsis, extracellular histones can act as damage-associated-molecular-patterns (DAMPs), reacting with different receptors, including Toll-like receptor (TLR) 2, TLR4, or TLR9 [5], and triggering activation of multiple pro-inflammatory pathways, including myeloid differentiation primary response 88 (MyD88), which can induce release of interleukin (IL)-6 and tumor necrosis factor (TNF) alpha, nuclear factor-κB (NF-κB), and activate NLRP3 inflammasome-dependent pathways [8]. Extracellular histones can also induce the release of IL-6 and TNF-alpha through TLR9 stimulation [8]. Extracellular histones can induce eryptotic death in human erythrocyte cells [9]. Additionally, extracellular histones can exert direct toxicity to endothelial cells and cause intestinal epithelium injury, impairing both the gut barrier [10] and blood–brain barrier function [11]. Furthermore, in septic patients, extracellular histones have been associated with coagulopathy, organ failure, and death [12]. It has also been reported that extracellular histone levels correlate with the Sequential Organ Failure Assessment (SOFA) score and survival in human sepsis [13].

Sodium-β-O-Methyl cellobioside sulfate (mCBS) is a small polyanion that interacts electrostatically with extracellular histones [14]. In vitro*,* mCBS treatment has been shown to reduce histone-induced cell cytotoxicity, histone-mediated hemolysis, red blood cell and platelet aggregation and degranulation [14]. mCBS also increased survival in a rat cecal ligation and puncture (CLP) sepsis model [14]. In a rat double-hit model of lipopolysaccharide (LPS)-induced acute lung injury, the administration of mCBS significantly decreased circulating histone concentration and improved acute lung injury and oxygenation [15].

We tested the hypothesis that neutralization of extracellular histones would reduce sepsis severity and improve outcome in a clinically relevant septic shock model in adult sheep.

## Methods

### Study setting

The study followed the EU Directive (2010/63/EU) for animal experiments and was approved by the local animal ethics committee (Protocol number 740N, Comité Ethique du Bien-Être Animal from the Université Libre de Bruxelles (ULB) in Brussels, Belgium). Experiments were performed in the Experimental Laboratory of Intensive Care of the ULB (LA1230406). The Animal Research: Reporting of In Vivo Experiments (ARRIVE) guidelines and Minimum Quality Threshold in Pre-Clinical Sepsis Studies (MQTiPSS) recommendations for translational research in sepsis were followed [16, 17].

### Experimental procedure

An ovine model of fecal peritonitis, adapted from previous experiments [18–21], was used, with 24 domestic female adult (6–8 months, 30–40 kgs) Suffolk sheep. Only female animals were used to facilitate access to bladder catheterization and increase homogeneity in the study cohort. On the day of the experiment, the animals were weighed, premedicated with an intramuscular mixture of 0.25 mg/kg midazolam and 20 mg/kg ketamine, and placed in the supine position. An 18G peripheral cannula was inserted into the cephalic vein to ensure vascular access.

After administration of an intravenous bolus of 30 μg/kg of fentanyl citrate, 1 mg/kg of propofol, and 0.1 mg/kg of rocuronium bromide, an 8-mm endotracheal tube was introduced. All the animals were sedated with 1.8–2.4% alveolar concentration of sevoflurane and a continuous intravenous infusion of morphine at a rate of 0.2–0.4 mg/kg/hour, with the optimal dose determined through repeated nociceptive tests, i.e., change in heart rate or blood pressure after nasal septum pinching. Rocuronium bromide was administered at a dose of 0.1 mg/kg/h for muscle paralysis. Hypoglycemia was avoided by giving a continuous infusion of a 20% glucose solution. A 60-cm-long plastic tube (inner diameter 1.8 cm) was inserted via the esophagus into the rumen to drain its content and to prevent rumen distension.

Mechanical ventilation was started in a volume-controlled mode (Primus, Dräger, Lübeck, Germany) using a tidal volume of 8 mL/kg, positive end-expiratory pressure of 5 cmH_2_O, a fraction of inspired oxygen of 30%, ratio of inspiratory time to expiratory time of 1:2, and a square-wave pattern. Respiratory rate was adjusted to maintain end-tidal carbon dioxide pressure (PetCO_2_) between 35 and 45 mmHg.

A 4.5 G arterial catheter was introduced into the left femoral artery under ultrasound guidance (Vivid E90, GE Machines, USA), connected to a pressure transducer and zeroed at the mid-thorax level. Pulse pressure variation (PPV) was automatically calculated from the arterial femoral signal using the formula [PPV = PP_max_ − PP_min_/(PP_max_ + PP_min_)/2], with PP being the pulse pressure (i.e., the difference between systolic and diastolic arterial pressures), continuously displayed (SC9000, Siemens, Munich, Germany), and exported to a recording station (Notocord-Hem 4.4, Notocord, France). A three-lumen central line catheter was inserted in the right jugular vein to provide fluid and drug infusions. In addition, an 8 Fr introducer was inserted into the left jugular vein, to introduce a 7.5 Fr Swan-Ganz catheter (CCO, Edwards LifeSciences, Irvine, California, USA) into the pulmonary artery. A left ventricular (LV) pressure catheter (5 Fr, Transonic® Europe BV, Elsloo, The Netherlands) was inserted into the left ventricle through the internal carotid artery and was connected to an ADV500 system (Transonic® Europe BV). A 14 Fr Foley catheter was inserted into the bladder and connected to a urine collection bag for monitoring of urine output.

A midline laparotomy and cecotomy were performed and 1.5 g/kg body weight of feces collected and stored; the cecum was then closed, and repositioned in the abdominal cavity. Two plastic tubes were placed for later introduction of the feces and peritoneal lavage. After abdominal surgery, the animals were positioned prone. Baseline measurements were taken after hemodynamic stabilization with normal arterial lactate levels. Feces were then injected into the abdominal cavity to induce peritonitis and sepsis.

**Fig. 1:**
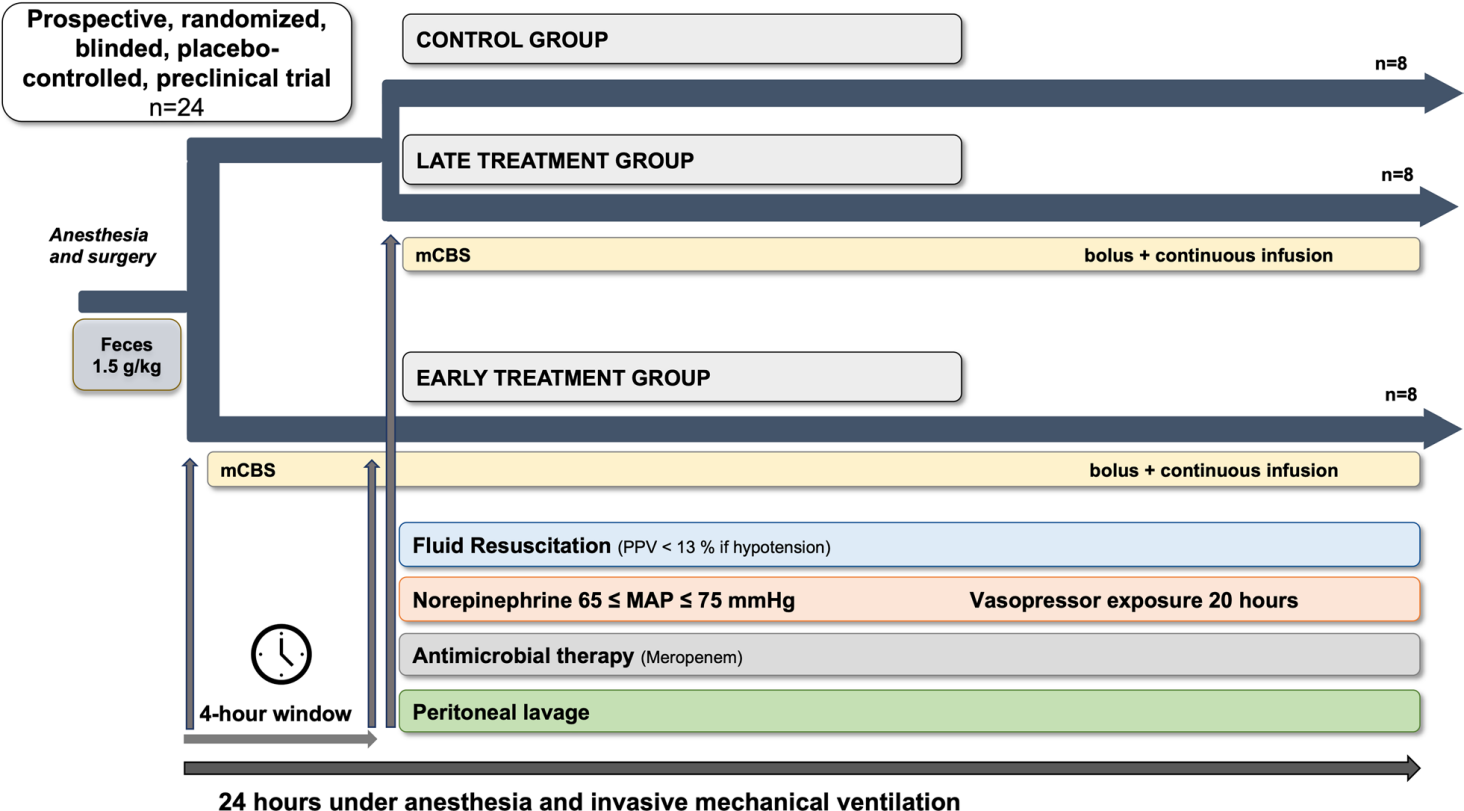
Protocol timeline

The timeline is shown in Fig. 1: during the first 4 h after feces injection, intravenous fluids were given at 2 mL/kg/h. Fluid resuscitation was then started with equal amounts of crystalloid (Plasmalyte, Baxter, USA) and colloid (Geloplasma, Fresenius Kabi, France) solutions, targeted to achieve a PPV < 13% in case of hypotension (mean arterial pressure [MAP] ≤ 65 mmHg). Intravenous norepinephrine was started if the MAP was ≤ 65 mmHg despite fluid administration and titrated to a maximum dose of 5 μg/kg/min.

Four hours after injection of the feces, meropenem was administered as an intravenous bolus of 20 mg/kg, followed by a continuous infusion of 2.5 mg/kg/hour until the end of the experiment. Peritoneal lavage was performed using four liters of warm (38 °C) saline through the abdominal tubes. Experiments were continued until spontaneous death or for 24 h, at which point the animals were euthanized under deep anesthesia with a bolus injection of 40 mL of 7.5% potassium chloride solution.

Data collection and blood sampling; multiplex cytokine magnetic bead panel assay; quantification of circulating H3.1 nucleosome concentration; sublingual microcirculation; and histological methods are described in Additional file 1.

### Experimental drug and randomization

Animals were randomized the day before the experiment to a control group, an early treatment group, or a late treatment group (*n* = 8 each). Investigators involved in the experiment were blinded to the group allocation of the animals. A 10 mg/mL concentration solution of mCBS (STC3141 provided by Grandpharma, Wuhan, China) was prepared and stored at 4 °C and infusion sets were prepared for each animal the day before the experiment by one of the investigators who was not involved in the experiment.

Immediately after feces injection, animals were given a first bolus injection of mCBS or saline (1 mg/kg) followed by a continuous infusion (1 mg/kg/h) for 4 h according to their randomized group. Four hours after feces injection, all the animals received a second bolus injection followed by a continuous infusion according to their randomized group until the end of the experiment.

The infusion sets for the three groups were therefore:

- Control: saline for both bolus injections and continuous infusions;
- Early treatment: mCBS for first bolus and continuous infusion, saline for second bolus and mCBS for second continuous infusion;
- late treatment: saline for first bolus and continuous infusion, mCBS for second bolus and continuous infusion.

### Statistical analysis

Given the absence of prior studies on large animals, an a priori convenient sample size of 8 animals per group was considered adequate for the study purpose. Statistical analysis was performed using Prism 9 (Version 9.1.2 (225). San Diego, CA, USA). Continuous variables are shown as mean ± standard deviation (SD) or median [25, 75% interquartile range (IQR)]. To estimate the effect of mCBS administration, a mixed-effects model with Greenhouse–Geisser correction was used. The effects of time and group, as well as interactions between group and time, were tested as fixed effects and animals were introduced as random effects. If there were significant differences, the two-stage linear procedure of Benjamini, Krieger, and Yekutieli, with individual variances, was used to compare the means of these variables for the groups at each time point. A post hoc analysis was performed excluding the two most severe animals from the control group. A chi-square test was used to detect differences in histological lung and kidney injury. The Pearson correlation coefficient was employed to analyze the relationship between H3.1 nucleosome levels and IL-6 levels, and norepinephrine dose. Differences in survival time were tested using a log-rank test for trend. A *p* value of < 0.05 was considered statistically significant.

## Results

### Hemodynamic and microcirculation findings

**Fig. 2:**
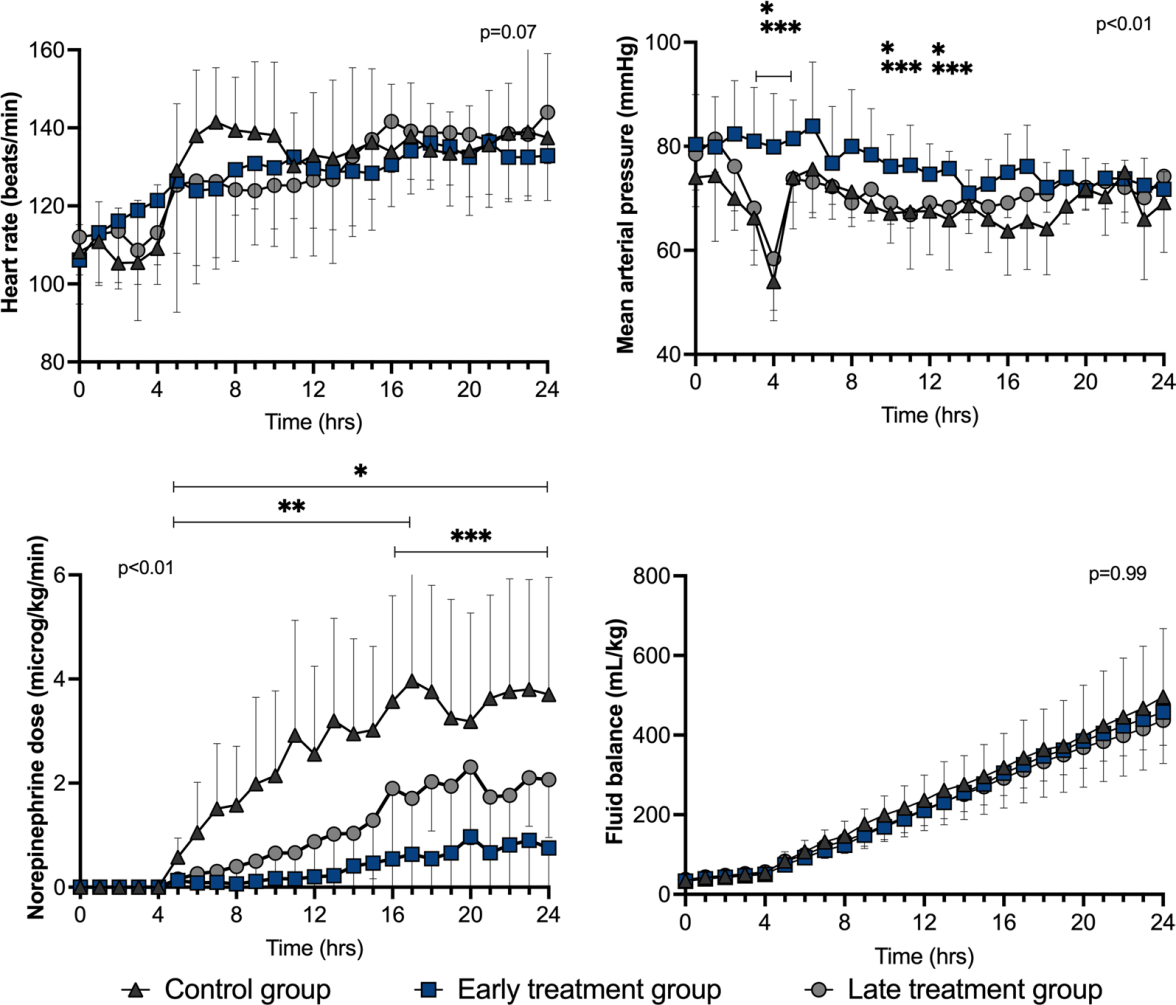
Heart rate, mean arterial pressure (MAP), norepinephrine requirements to maintain MAP between 65 and 75 mmHg, and fluid balance in the three groups during the study period. Values are expressed as mean ± SD. *p* value given for overall interaction Time × Group. *p < 0.05 between early treatment and control groups in case of overall interaction. **p < 0.05 between late treatment and control groups in case of overall interaction. ***p < 0.05 between early treatment and late treatment groups in case of overall interaction

There were no statistically significant differences in measured variables among groups at baseline (Additional file 1: Table S1). MAP decreased rapidly after feces injection in the control and late treatment groups, but not in the early treatment group (Fig. 2). Significantly lower norepinephrine doses were required to maintain MAP between 65 and 75 mmHg in the early treatment and late treatment groups than in the control group (Fig. 2), despite a similar fluid balance among groups (Fig. 2).

**Fig. 3:**
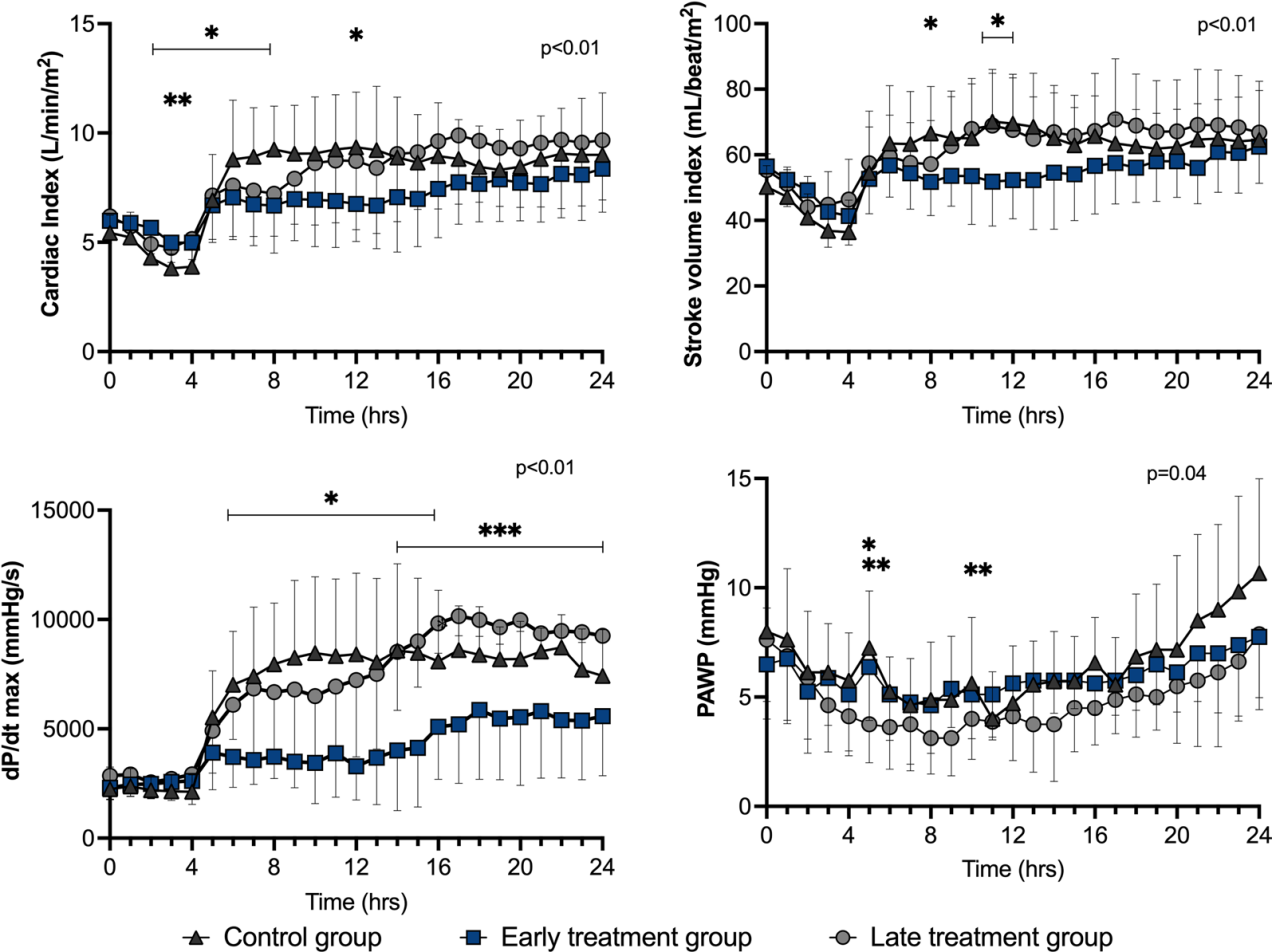
Cardiac hemodynamic variables during the study period in the three groups. Values are expressed as mean ± SD. PAWP: pulmonary artery wedge pressure; *p* value given for overall interaction (Time × Group). *p < 0.05 between early treatment and control groups in case of overall interaction. **p < 0.05 between late treatment and control groups in case of overall interaction. ***p < 0.05 between early treatment and late treatment groups in case of overall interaction

**Fig. 4:**
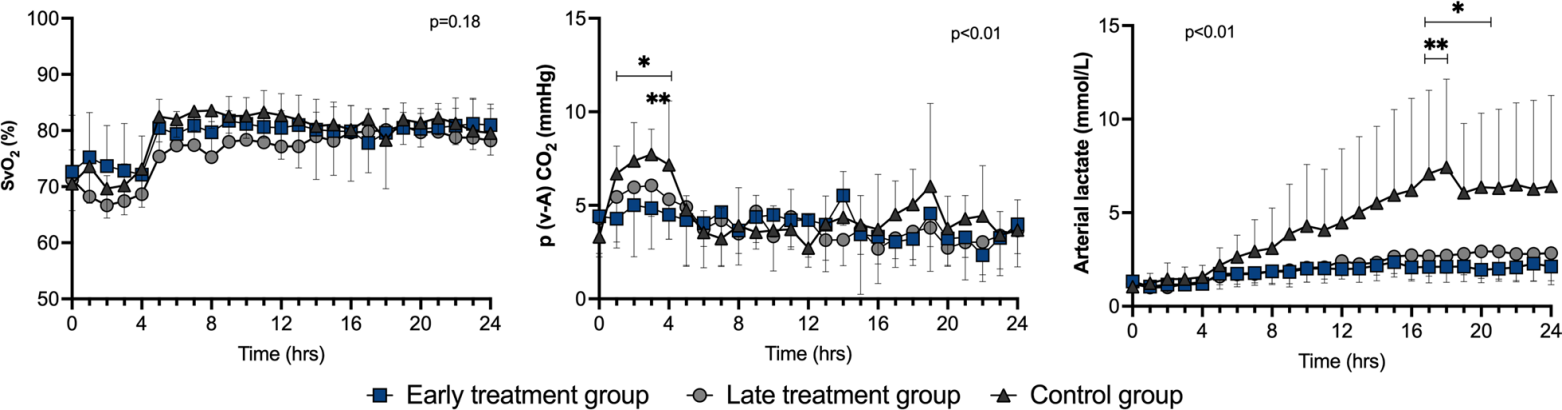
Oxygenation indexes and arterial lactate levels during the study period in the three groups. Values are expressed as mean ± SD. SvO_2_: mixed venous oxygen saturation; P(v-a) CO_2_: veno-arterial carbon dioxide tension difference. *p* value given for overall interaction (Time × Group). *p < 0.05 between early treatment and control groups in case of overall interaction. **p < 0.05 between late treatment and control groups in case of overall interaction

After initial resuscitation (T4), there were no relevant statistically significant differences in heart rate, cardiac output, stroke volume, pulmonary artery wedge pressure (PAWP), mixed venous oxygen saturation (SvO_2_) or veno-arterial carbon dioxide tension difference (P(v-a) CO_2_) among groups (Figs. 2, 3 and 4). The LV d*P*/d*t*_max_ was lower in the early treatment than in the late treatment group from T14 to T24 and than in the control group from T6 to T16 (Fig. 3). Arterial blood lactate was lower in the treated groups than in the control group from T17 (Fig. 4).

The proportion of perfused vessels was larger in the treated groups at T6 (two hours after resuscitation) than in the control group; there were no statistically significant differences among groups after T6 (Additional file 1: Figure S1).

### Organ function

**Fig. 5:**
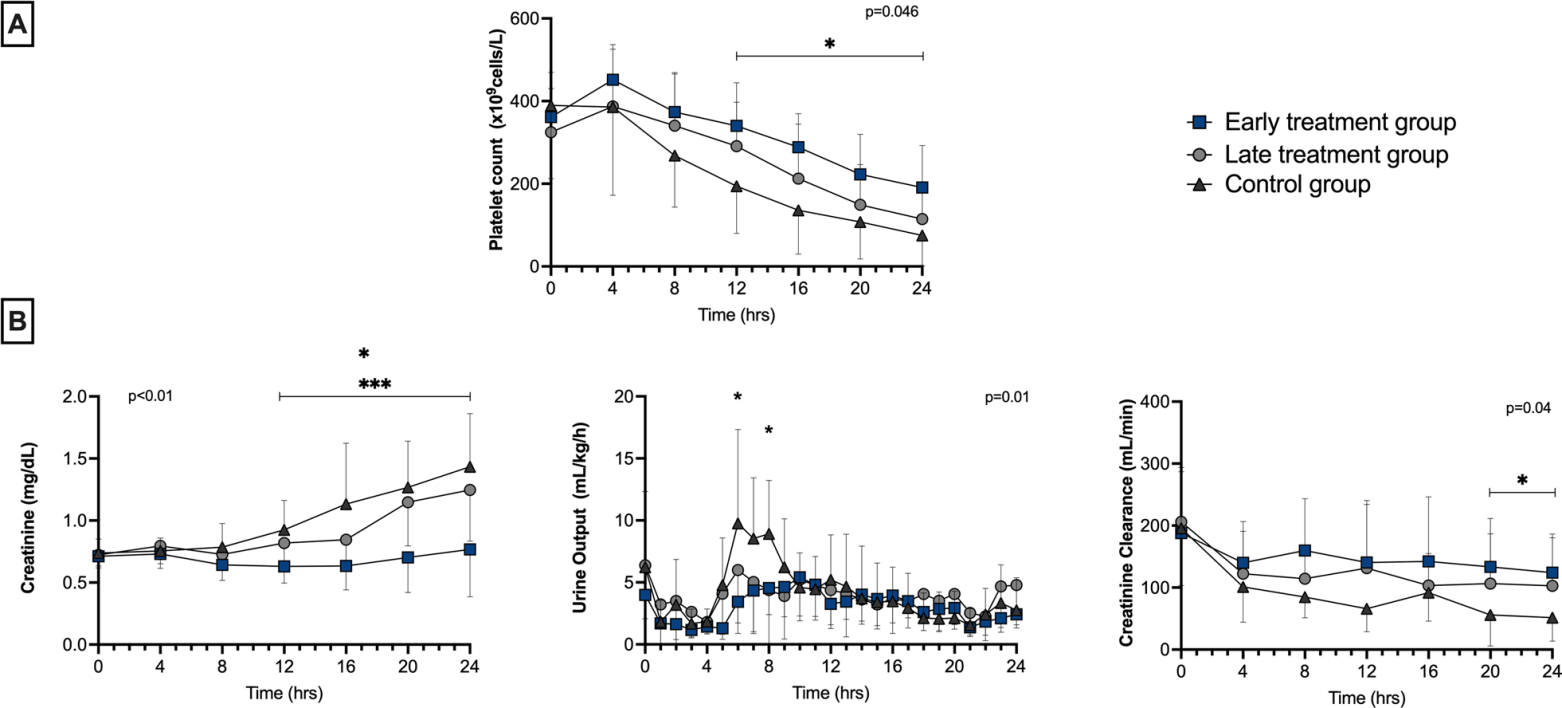
Platelet count (**A**), and kidney function (**B**) during the study period in the three groups. Values are expressed as mean ± SD. *p* value given for overall interaction (Time × Group). *p < 0.05 between early treatment and control groups in case of overall interaction. ***p < 0.05 between early treatment and late treatment groups in case of overall interaction

The platelet count was significantly higher in the early treatment group than in the control group (from 12 h) (Fig. 5). Creatinine levels remained within normal ranges in the early treatment group throughout the study period and were significantly lower than those in the late treatment and control groups from 12 h (Fig. 5). Urine output was similar among groups, but creatinine clearance was significantly higher in the early treatment group at T20 and T24 than in the control group (Fig. 5).

On histological analysis (performed only in the last 16 animals), there was no evidence of acute glomerular ischemia in the early treatment group (0/5); it was present in 1/6 animals in the late treatment group and 3/5 in the control group. There were no signs of acute tubular necrosis in the early treatment group (0/5), but it was present in 2/6 of the late treatment animals and 2/5 of the control animals (Additional file 1: Figure S2).

The PaO_2_/FiO_2_ ratio, the respiratory system compliance, the lung wet/dry ratio, and the acute lung injury score were similar among groups (Additional file 1: Figures S2 and S3). There were no clinically relevant differences in other measured biological variables (Additional file 1: Figures S4 and S5).

### Systemic circulating nucleosomes and cytokines

**Fig. 6:**
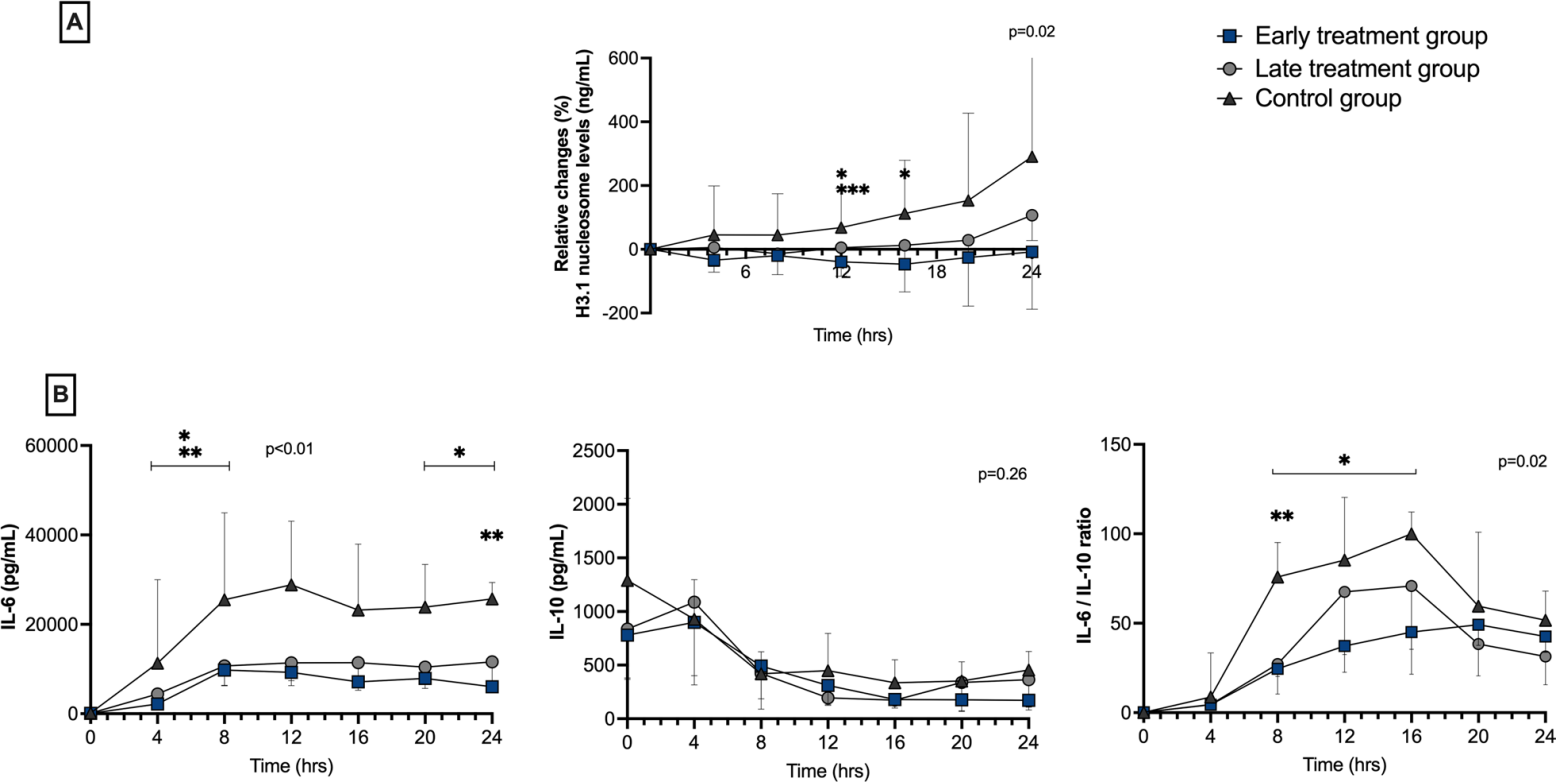
Changes in H3.1 nucleosome levels from baseline (ng/mL) (**A**) and measured cytokine levels (**B**) at different timepoints in the three groups. Values given are median and interquartile range; IL: interleukin. *p* value given for overall interaction (Time × Group). *p < 0.05 between early treatment and control groups in case of overall interaction. **p < 0.05 between late treatment and control groups in case of overall interaction. ***p < 0.05 between early treatment and late treatment groups in case of overall interaction

Changes in circulating H3.1 nucleosome levels from baseline values differed between the early and control groups at T12 and T16, and between the early and late treatment groups at T12 (Fig. 6). Circulating plasma levels of IL-6 were lower in the treatment groups than in the control group from T4 to T8 and at T24 and the IL-6-to-IL-10 ratio was lower in the early group from T8 to T16 and in the late group at T8 (Fig. 6). The Pearson correlation coefficient (r) between IL-6 levels and H3.1 nucleosomes levels was 0.33 (95% CI 0.19 to 0.46; *p* < 0.01), and 0.63 (95% CI 0.52 to 0.71; *p* < 0.01) between H3.1 nucleosome levels and norepinephrine dose.

### Survival

Two (25%) of the animals in the control group died before 24 h, at T12 and T18, whereas all the animals in the treatment groups survived. The differences between groups in hemodynamic, oxygenation, renal function and inflammation variables were similar to those mentioned earlier after excluding these two animals from the control group (Additional file 1: Figures S6–S10).

## Discussion

We used a clinically relevant ovine model of septic shock secondary to fecal peritonitis; treatment was quite complete and included fluid resuscitation, antibiotic therapy, peritoneal lavage, and vasopressor administration. In this context, our main findings reveal that mCBS treatment significantly: (1) blunted the increases in H3.1 nucleosome levels observed in the control group; (2) reduced vasopressor requirements to maintain mean arterial pressure; (3) decreased arterial lactate levels and reduced organ failure; and (4) attenuated the increase in the pro-inflammatory response.

mCBS treatment significantly decreased changes in H3.1 nucleosome levels in the two treatment groups, although data have suggested that mCBS may neutralize extracellular histones, but not nucleosomes [14]. This suggests an interruption in the amplification cascade of histone-induced nucleosome release, as histones have been shown to possess direct cellular toxicity, which results in a vicious cycle of further cell death and nucleosome release that could be prevented by mCBS treatment [22]. As nucleosome levels have been shown to be associated with severity in septic shock patients, and to be higher in non-survivors compared to survivors upon admission and throughout the first week of sepsis [23, 24], further studies are needed to determine whether nucleosome levels could serve as a guide for mCBS therapy.

Early mCBS administration was associated with less severe septic shock, with preserved MAP during the first 4 h and lower norepinephrine requirements in the two treatment groups. This beneficial effect might be related to preserved endothelial function, as histones are known to have direct cytotoxic effects on endothelial cells in vitro, and small polyanions like mCBS can prevent these effects [14, 25, 26]. Furthermore, the reduced catecholamine exposure could also be beneficial by reducing adrenergic stimulation to the cardiovascular system, which has been described as contributing to septic cardiomyopathy, as well as reducing harmful non-hemodynamic effects, such as immune dysregulation [27–29]. In particular, norepinephrine can dysregulate the immune response and compromise host defense mechanisms, which, during experimental sepsis, was associated with increased bacterial dissemination compared to vasopressin [29].

mCBS has been shown to prevent the cytotoxic effects of extracellular histones on endothelial cells in vitro [14, 25, 26], an effect that was not assessed in the current study. mCBS may also limit the deleterious effects of extracellular histones on cardiomyocytes [30, 31]. Circulating histone levels (> 75 μg/ml) have been associated with new onset LV dysfunction and arrhythmias in septic shock patients without prior cardiac disease [32]. However, our model did not show impaired contractility, assessed by d*P*/d*t*_max_, which might be due to the relatively short observation period.

The majority of the favorable effects attributable to the use of mCBS were noted predominantly in the early treatment group. However, delayed administration was associated with reduced norepinephrine requirements and a decrease in arterial lactate levels. Notably, there was no discernible impact of late treatment on platelet count or renal function. The influence of this therapeutic approach on patient outcomes within real-world clinical settings, where treatment initiation often occurs later than in controlled experimental studies, has yet to be thoroughly explored. Further investigation is warranted to elucidate the potential benefits of mCBS in the context of clinical practice.

mCBS treatment may be associated with improved tissue perfusion as suggested by preserved sublingual microcirculation at T6 in the treated groups, lower lactatemia, and lower creatinine levels than in control animals. This could be partly attributed to improvements in coagulation and thrombosis. Extracellular histones are associated with coagulopathy during sepsis and can contribute to the formation of neutrophil extracellular traps (NETs), which promote macro/micro-thrombosis and immuno-thrombosis [12, 33]. The reduced changes in circulating nucleosome levels, a marker of NET formation, support this hypothesis. Additionally, a higher platelet count was observed in animals with early mCBS administration, consistent with data indicating that extracellular histones promote red blood cell and platelet aggregation in vitro [33]. However, other coagulation parameters were similar across groups, despite some non-clinically relevant differences early during the experiment.

Sepsis-associated acute kidney injury (AKI) was prevented in the early treatment group, as indicated by lower creatinine levels and fewer pathological findings than in the control animals. This may be related to reduced norepinephrine exposure, reduced toxicity, or to reduced TLR stimulation via extracellular histones on tubular cells, since TLR antagonists may limit local inflammation and attenuate AKI [5, 34, 35]. Although extracellular histones can mediate lung injury [36], we did not observe any protective effect of treatment on lung pathology or function, likely related to a preserved lung function in the actual model.

mCBS limited the increase in IL-6 levels and in the IL-6-to-IL-10 ratio observed in the control group, a result in line with the pro-inflammatory effects of extracellular histones. Histones can activate neutrophils to form NETs [36], can increase tumor necrosis factor (TNF)-α, IL-6, and IL-10, through a TLR4-dependent pathway [37], and can activate a MyD88-dependent pathway through TLR2 and TLR4 [5]. Histones can interact with TLR2 expressed on T cells, inducing STAT3 phosphorylation and Th17 polarization. Furthermore, mCBS was able to abolish the interleukin-17 production induced by histones [38].

Extracellular histones have recently been shown to increase monocyte distribution width (MDW) index [39]; however, we only assessed the white blood cell count. The reduction in the pro-inflammatory response may potentially alter the innate immune response, and this should be assessed over a longer observation period [22, 40].

There was a tendency for improved survival in mCBS-treated animals, with 2 deaths in the control group and none in the treated animals. This trend might be attributed to the factors mentioned above, such as improved coagulation, inflammatory response, or endothelial function. Assessment of effects on mortality in large animal studies can, however, be challenging due to small sample sizes. In a rat CLP model, mCBS administration was associated with improved survival over a 20-h observation period [14]. Improved survival has also been observed with other anti-histone agents in sepsis: in mice, deoxyribonuclease administration attenuated sepsis-induced organ damage and improved survival in LPS and CLP sepsis models [41–43]. In a mouse LPS model, an antibody targeting histone H3 decreased circulating IL-1β and TNF-α concentrations, reduced acute lung injury, and improved survival [44]. However, no study testing extracellular histone neutralization has been performed in a large animal model of peritonitis-induced septic shock that meets preclinical research recommendations for clinical relevance and external validity regarding management, with administration of fluids, source control (peritoneal lavage and antibiotic therapy), and early vasopressor administration [17, 45].

The present study was randomized, blinded, and placebo-controlled, and all the interventions were protocolized. In particular, the fluid protocol used in the study was based on a dynamic assessment of fluid responsiveness, targeting a PPV < 13% when hypotension occurred [46]. Moreover, all the catheters were introduced percutaneously under ultrasound guidance, limiting tissue inflammation related to surgery.

There are, however, several limitations of the study. First, we included young healthy animals, different to the clinical setting where patients usually have several comorbidities. Second, *post-mortem* analyses were not available for all the animals. Third, circulating histones were not assessed in our study, and only H3.1 nucleosome levels, which are biomarkers of NETosis, were reported [47]. However, it is important to note that circulating histones have been shown to induce NET formation, and nucleosomes may serve as an indirect marker of histone neutralization and NET formation [47, 48]. Fourth, we did not assess endothelial function using biomarkers or other techniques. Lastly, a spectrometry-based method for quantifying circulating histones could have been employed to provide evidence of extracellular histone neutralization [49].

A phase I study in healthy volunteers showed that mCBS administration was safe, with a linear pharmacokinetic relationship with increasing doses. Its half-life is between 2.5 and 3.3 h, and it is mainly excreted through the kidneys (data provided by Grand Pharma). Based on the phase 1 study, the current experiment, and results from a phase IIa study assessing safety in patients with COVID-19 (NCT04880694), multicenter trials of mCBS in sepsis are currently ongoing.

## Conclusions

In this clinically relevant septic shock model, mCBS treatment modulated the inflammatory response as shown by differences in systemic IL-6 and changes in H3.1 nucleosome levels. This effect was associated with an increase in vascular tone, some improvement in tissue perfusion, and less sepsis-associated AKI. Targeting extracellular histones could open a new therapeutic approach for sepsis.

### Supplementary Information

**Additional file 1.** Additional methods, microcirculation results, histological analysis, and secondary analysis with dead animals excluded.

## Data Availability

The datasets used during the current study are available from the corresponding author on reasonable request.
