# Supplementary material for: Neutralization of extracellular histones by sodium-Β-O-methyl cellobioside sulfate in septic shock
Source: Crit Care. 2023 Nov 24;27:458. doi: 10.1186/s13054-023-04741-x (PMC10675855; doi:10.1186/s13054-023-04741-x)
Supplement: Supplementary file 1 — Additional file 1. Additional methods, microcirculation results, histological analysis, and secondary analysis with dead animals excluded. [file 13054_2023_4741_MOESM1_ESM.docx]

**Neutralization of Extracellular Histones by Sodium-Β-O-Methyl Cellobioside Sulfate in Septic Shock**

Bruno Garcia^1,2^, Fuhong Su^1^, Laurence Dewachter^3^, Yong Wang^4^, Ning Li^4^, Myriam Remmelink^5^, Marie Van Eycken^5^, Amina Khaldi^1^, Raphaël Favory^2^, Antoine Herpain^1^, Anthony Moreau^1,6^, Alexander Moiroux-Sahraoui^1^, Francesca Manicone^1^, Filippo Annoni^1,6^, Lin Shi^4^, Jean-Louis Vincent^1^, Jacques Creteur^1^, Fabio S. Taccone^1,6^

^1^Experimental Laboratory of the Department of Intensive Care, Erasme Hospital, Université Libre de Bruxelles, Brussels, Belgium

^2^Department of Intensive Care, Centre Hospitalier Universitaire de Lille, Lille, France

^3^Laboratory of Physiology and Pharmacology, Université Libre de Bruxelles, Brussels, Belgium

^4^Grand Pharma (China) Co., Ltd, Wuhan, China

^5^Pathology Laboratory, Erasme Hospital, Hôpitaux Universitaires de Bruxelles, Université Libre de Bruxelles, Brussels, Belgium

^6^Department of Intensive Care, Erasme Hospital, Hôpital Universitaire de Bruxelles, Université Libre de Bruxelles, Brussels, Belgium

**Corresponding author**: Jean-Louis VINCENT, MD, PhD. Department of Intensive Care, Erasme Hospital, 1070 Brussels, Belgium. E-mail: jlvincent@intensive.org

**TABLE OF CONTENTS:**

[METHODS 4](#_Toc150075611)

[Monitoring, data collection and blood sampling 4](#_Toc150075612)

[Multiplex cytokine magnetic bead panel assay 5](#_Toc150075613)

[Quantification of circulating levels of H3.1 nucleosomes 5](#_Toc150075614)

[Sublingual microcirculation 5](#_Toc150075615)

[Autopsy 6](#_Toc150075616)

[Table S1. Baseline characteristics 7](#_Toc150075617)

[Figure S1. Proportion of perfused vessels 8](#_Toc150075618)

[Figure S2. Histological analysis 9](#_Toc150075619)

[Figure S3. Pulmonary function 10](#_Toc150075620)

[Figure S4 Base excess, white and red blood cell counts, hemoglobin concentration during study period 11](#_Toc150075622)

[Figure S5. Aspartate aminotransferase (AST), alanine aminotransferase (ALT), and coagulation parameters during the study period 12](#_Toc150075623)

[Figure S6. Heart rate, mean arterial pressure (MAP), norepinephrine requirements to maintain MAP between 65 and 75 mmHg, and fluid balance in the three groups during the study period with the two most severe animals excluded from the control group. 13](#_Toc150075624)

[Figure S7. . Cardiac hemodynamic variables during the study period in the three groups with the two most severe animals excluded from the control group. 14](#_Toc150075625)

[Figure S8. Oxygenation indexes and arterial lactate levels during the study period in the three groups with the two most severe animals excluded from the control group. 15](#_Toc150075626)

[Figure S9. Platelet count (A) and kidney function (B) during the study period in the three group with the two most severe animals excluded from the control group. 16](#_Toc150075627)

[Figure S10. Changes in H3.1 nucleosome levels from baseline (ng/mL) (A) and measured cytokine levels (B) at different timepoints in the three groups with the two most severe animals excluded from the control group. 17](#_Toc150075628)

[ONLINE DATA SUPPLEMENT - REFERENCES 18](#_Toc150075629)

METHODS

An ovine model of fecal peritonitis, adapted from previous experiments (1–4), was used, with 24 domestic female adult (6-8 months, 30-40 kgs) Suffolk sheep. According to the 3R principles (replacement, reduction and refinement), some of the animals had been used as controls in a previous experimental study (4). Only females were used to facilitate access to bladder catheterization and increase homogeneity. Three groups were considered in the current protocol: a control group (n=8), an early-treatment group (n=8), and a late-treatment group (n=8).

Monitoring, data collection and blood sampling

Hemodynamic parameters, including HR (heart rate), MAP (mmHg), pulmonary artery pressure (PAP, mmHg), and LV dP/dT_max_ were continuously displayed (SC9000, Siemens, Munich, Germany) and exported to an A/D recording station (Notocord-Hem 4.4, Notocord, France). Variables were referenced to the mid-chest level, and obtained at end expiration. Core temperature (°C), cardiac output (L/min) (Vigilance II; Edwards Lifesciences, California, USA), minute volume (mL), plateau pressure (mmHg), expiratory tidal volume (mL), and end-tidal carbon dioxide pressure (mmHg) were continuously monitored. Cardiac index (L/min/m^2^), and stroke volume index (mL/m^2^) were calculated using standard formulas.

Urine output was monitored hourly. Arterial and mixed central venous blood gas samples were obtained every hour. Additional arterial samples were obtained at baseline and T4, T8, T12, T16, T20, and T24 hours after sepsis induction for later determination of blood creatinine, interleukin (IL)-6 and (IL)-10 levels, and circulating H3.1 nucleosome levels. They were sampled in EDTA-syringes and centrifuged at 3000 rounds per minute for 15 minutes, then immediately frozen at -80°C until analysis.

Multiplex cytokine magnetic bead panel assay

Circulating levels of IL-6 and IL-10 in systemic arterial plasma samples were determined using a cytokine magnetic bead panel assay (MILLIPLEX^MAP^ Ovine Cytokine Multiplex Assay, Merck, Germany), according to the manufacturer’s instructions. Plasma cytokine concentrations were obtained by referring to a standard curve realized in parallel. Results represent the mean value of two separate measurements performed in duplicate at each time point.

Quantification of circulating levels of H3.1 nucleosomes

Circulating levels of H3.1 nucleosomes in systemic plasma samples were determined using a Nu.Q® H3.1 ELISA sandwich assay (1001-01-03, Volition, Isnes, Belgium), according to the manufacturer’s instructions. Plasma concentrations of H3.1 nucleosomes were obtained by reading absorbance values at 450 nm referring to a standard curve performed in parallel. Results represent the averaged values of two separate measurements performed in duplicate and are reported as relative changes from the baseline value.

Sublingual microcirculation

At T0, T6, T12, T18 and T24, the microvascular network of the sublingual area was studied using an incident dark field (IDF) camera with a 5x objective providing 167x magnification. The device was applied without pressure and images from five areas were recorded with a minimum duration of 5 seconds each. The images were then stored by random number designation for further automatic analysis of the percentage of perfused vessels using specific software (Analysis Manager, V3, Braedius, The Netherlands) (3).

Autopsy

After spontaneous death or death by euthanasia of the animals, autopsy of the lung and kidney was rapidly performed. The middle lobe of the right lung and the left kidney were carefully removed and rinsed with saline then fixed overnight in 4% formalin and embedded in paraffin. Lung (5) and kidney (6) injury were assessed by two independent pathologists in duplicate in a blinded manner. Briefly, hematoxylin and eosin-stained lung sections were analyzed for neutrophil infiltration, airway epithelial cell damage, interstitial edema, hyaline membrane formation, and hemorrhage. Each criterion was scored on a scale of 0 to 4, where 0 = normal, 1 = minimal change, 2 = mild change, 3 = moderate change, and 4 = severe change. The total acute lung injury score was the sum of the values for the 5 criteria. For the kidney, the presence of glomerular ischemia and acute tubular necrosis were noted.

Part of the right middle lung lobe was heated to 200°C for 24 hours and then weighed again to calculate the wet/dry ratio.

Table S1. Baseline characteristics

| Variables | Control  (N = 8) | Early-treatment (N = 8) | Late-treatment  (N = 8) | *P* value |
| --- | --- | --- | --- | --- |
| Body weight (kg) | 36 ± 1 | 35 ± 1 | 37 ± 1 | 0.36 |
| Temperature (°C) | 38.2 ± 0.3 | 38.1 ± 0.2 | 38.7 ± 0.2 | 0.11 |
| Heart rate (beats/min) | 108 ± 2 | 106 ± 4 | 112 ± 3 | 0.47 |
| MAP (mmHg) | 74 ± 2 | 80 ± 3 | 79 ± 2 | 0.24 |
| mPAP (mmHg) | 19 ± 3 | 15 ± 1 | 14 ± 1 | 0.21 |
| PaO_2_/FiO_2_ ratio | 303 ± 27 | 321 ± 22 | 337 ± 13 | 0.56 |
| Arterial lactate (mmol/L) | 1.1 ± 0.1 | 1.3 ± 0.2 | 1.2 ± 0.1 | 0.29 |

Data are given as mean ± standard deviation. MAP: Mean arterial pressure; mPAP: mean pulmonary artery pressure

Figure S1. Proportion of perfused vessels

Values are mean ± SD.

* *P*<0.05 between early-treatment group and control group in case of overall interaction

** *P* <0.05 between late-treatment group and control group in case of overall interaction

Figure S2. Histological analysis

1. **Lung wet / dry ratio and Acute Lung Injury Score in the three groups**
2. **Kidney histology: frequency of glomerular ischemia and acute tubular necrosis in the three groups**

Figure S3. Pulmonary function

Values are expressed as mean ± SD.

* p<0.05 between early-treatment and control groups in case of overall interaction

***p<0.05 between early-treatment and late-treatment groups in case of overall interaction


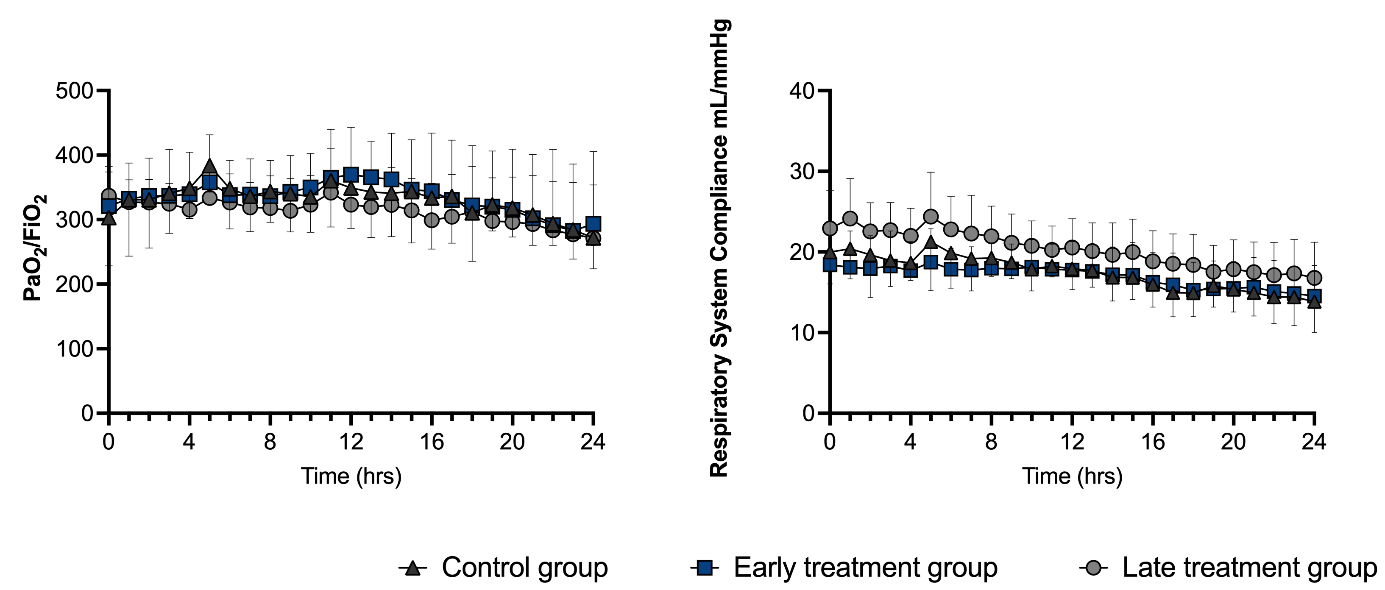


Figure S4 Base excess, white and red blood cell counts, hemoglobin concentration during study period


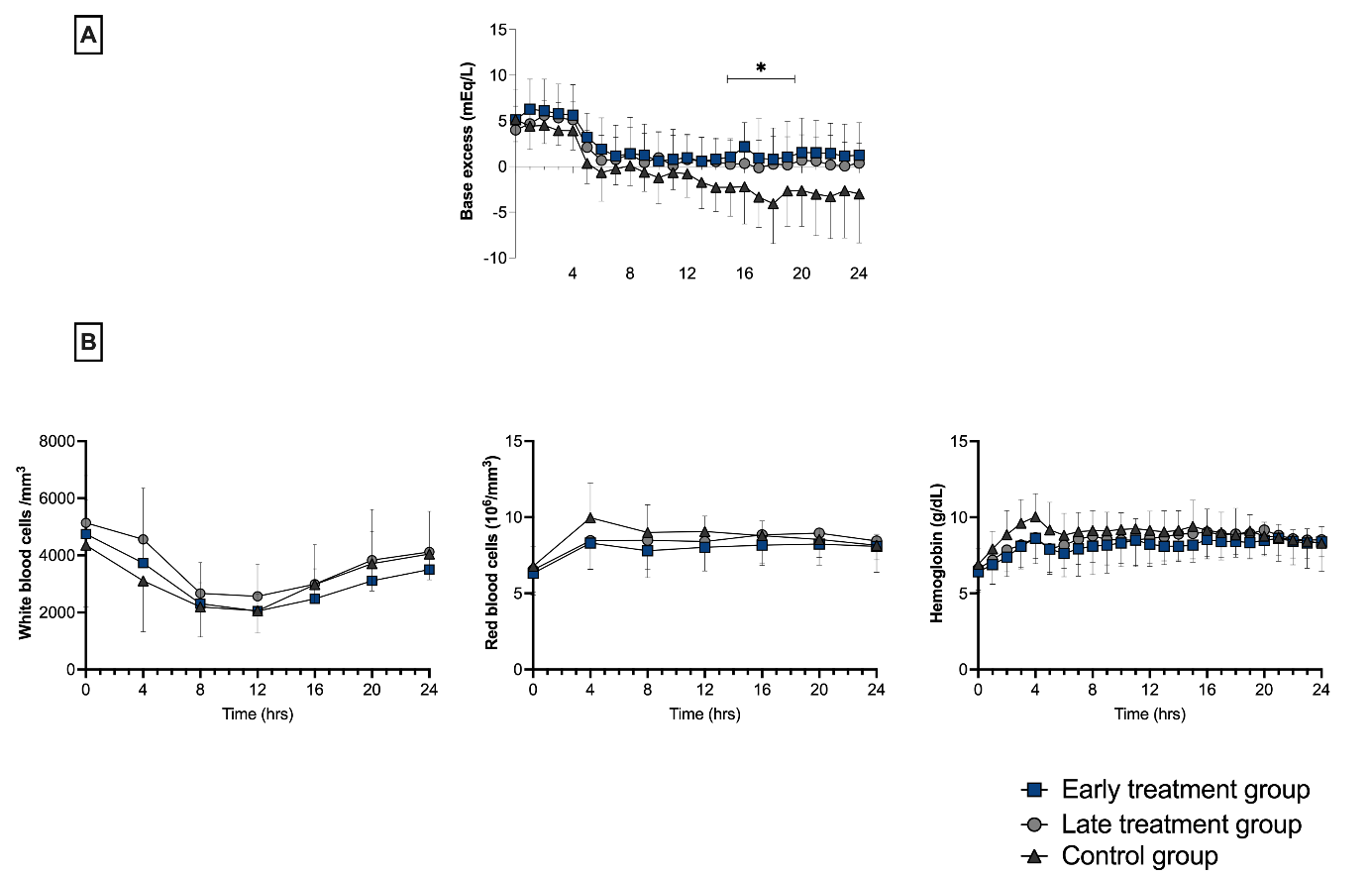


1. **Base excess**
2. **White and red blood cell counts, and hemoglobin concentration**

Values are mean ± SD.

* p<0.05 between early-treatment and control groups in case of overall interaction

Figure S5. Aspartate aminotransferase (AST), alanine aminotransferase (ALT), and coagulation parameters during the study period


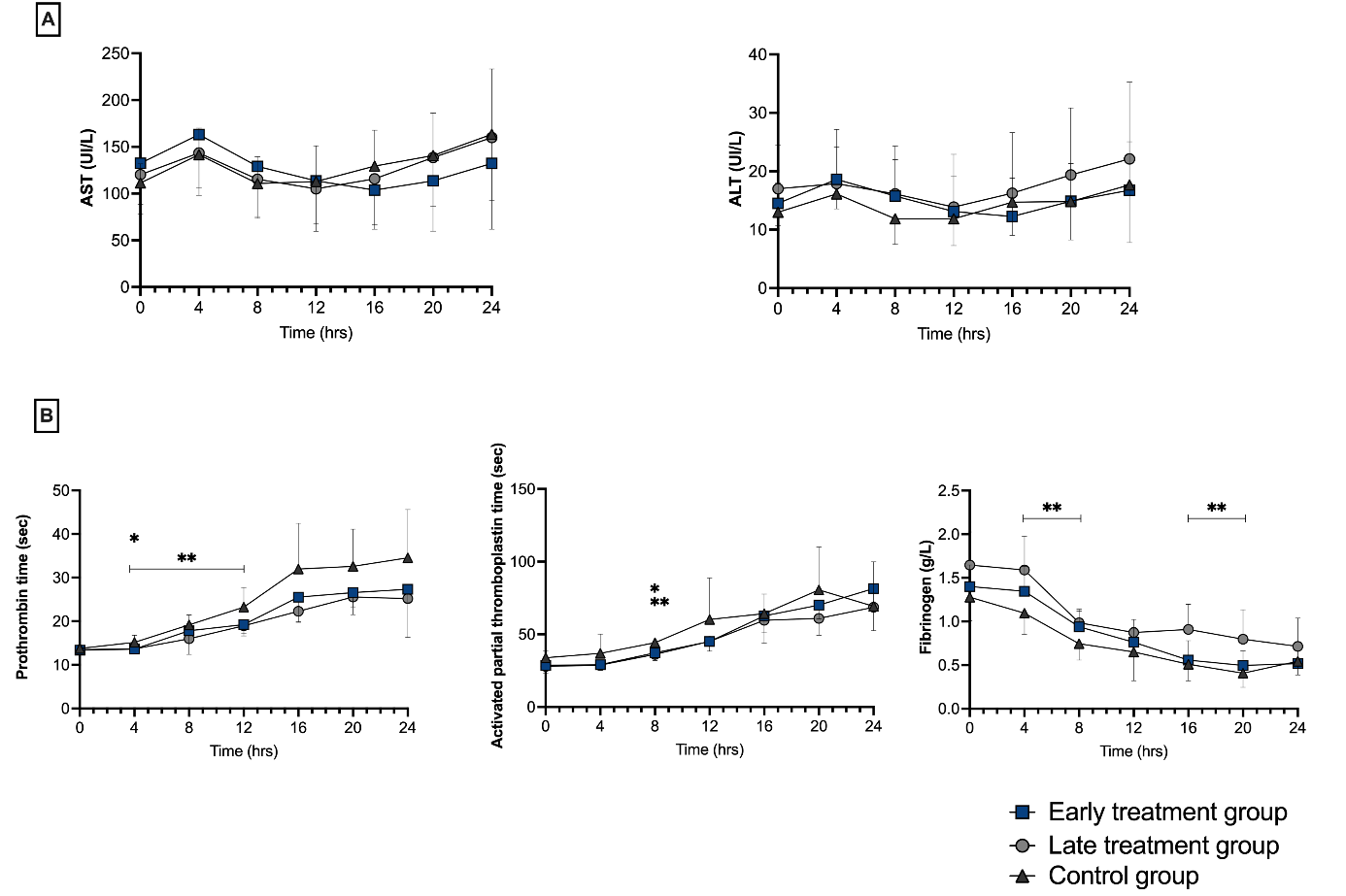


1. **Aspartate aminotransferase (AST), alanine aminotransferase (ALT),**
2. **Coagulation parameters**

Values given are mean ± SD.

* p<0.05 between early-treatment and control groups in case of overall interaction

** p<0.05 between late-treatment and control groups in case of overall interaction

Figure S6. Heart rate, mean arterial pressure (MAP), norepinephrine requirements to maintain MAP between 65 and 75 mmHg, and fluid balance in the three groups during the study period with the two most severe animals excluded from the control group.

Values are expressed as mean ± SD.

* p<0.05 between early-treatment and control groups in case of overall interaction

***p<0.05 between early-treatment and late-treatment groups in case of overall interaction


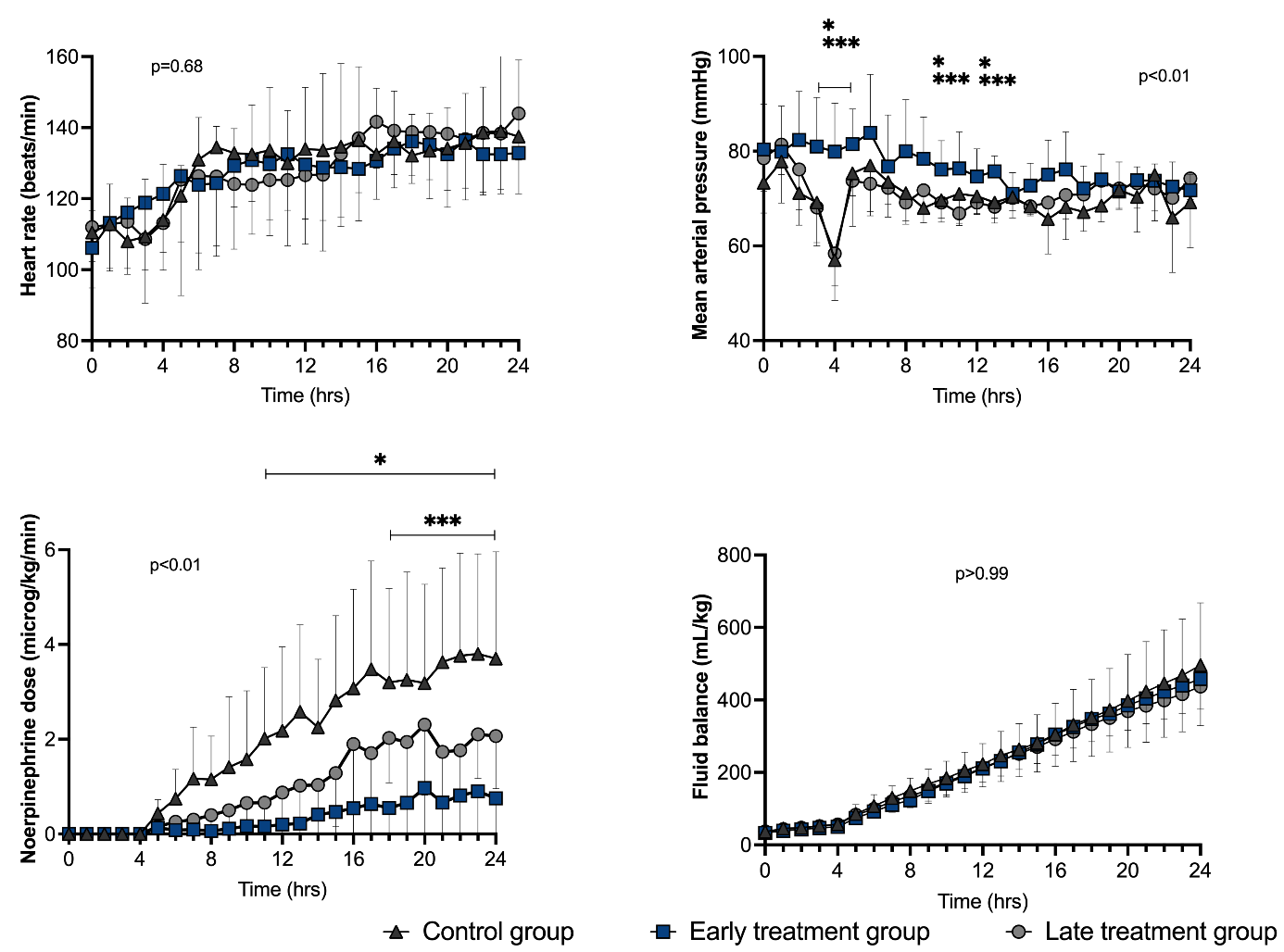


Figure S7. . Cardiac hemodynamic variables during the study period in the three groups with the two most severe animals excluded from the control group.

Values are expressed as mean ± SD. PAWP: pulmonary artery wedge pressure;

* p<0.05 between early-treatment and control groups in case of overall interaction

***p<0.05 between early-treatment and late-treatment groups in case of overall interaction


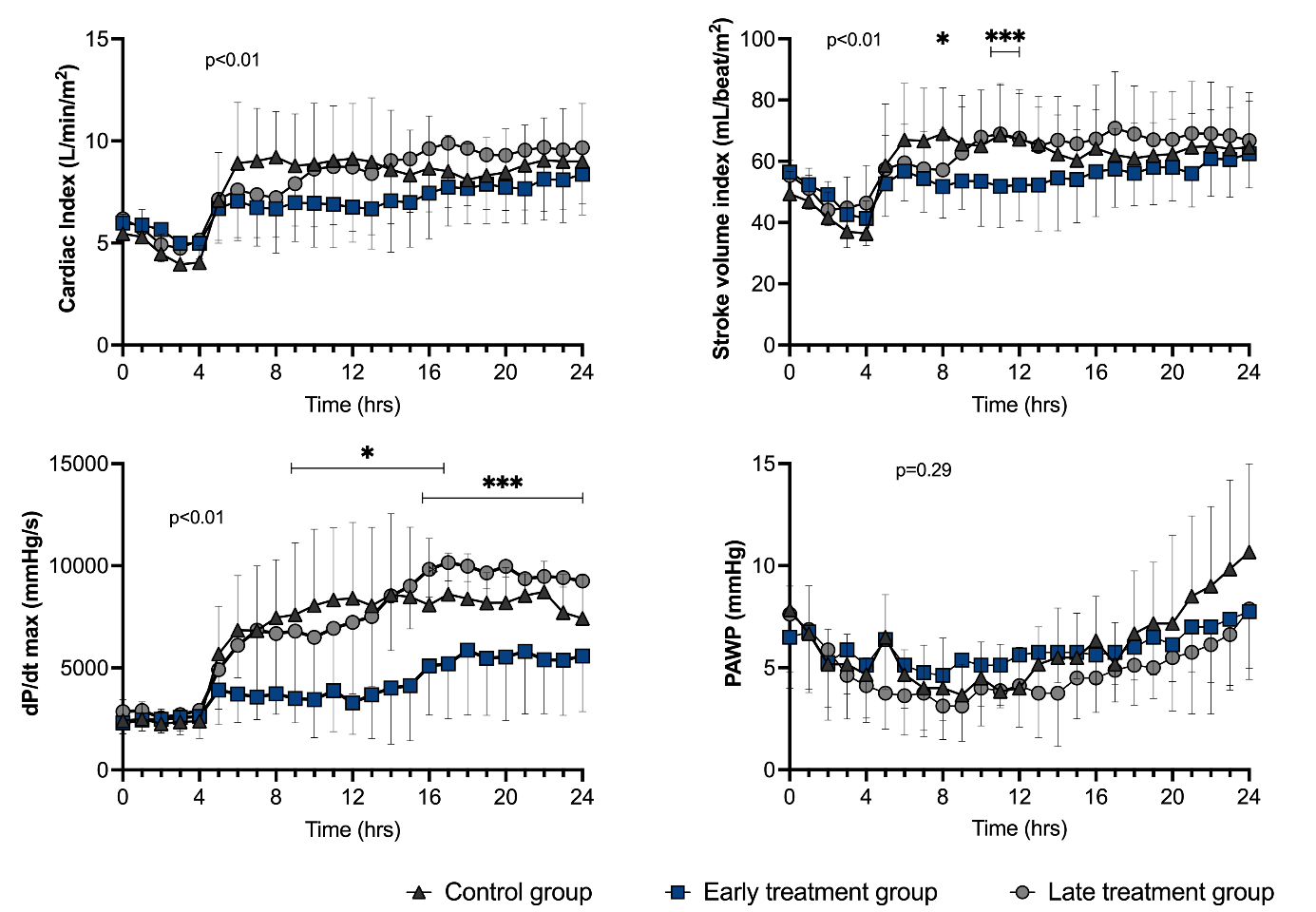


Figure S8. Oxygenation indexes and arterial lactate levels during the study period in the three groups with the two most severe animals excluded from the control group.

Values are expressed as mean ± SD.

SvO_2_: mixed venous oxygen saturation; P(v-a) CO_2_: veno-arterial carbon dioxide tension difference

* p<0.05 between early-treatment and control groups in case of overall interaction

** p<0.05 between late-treatment and control groups in case of overall interaction


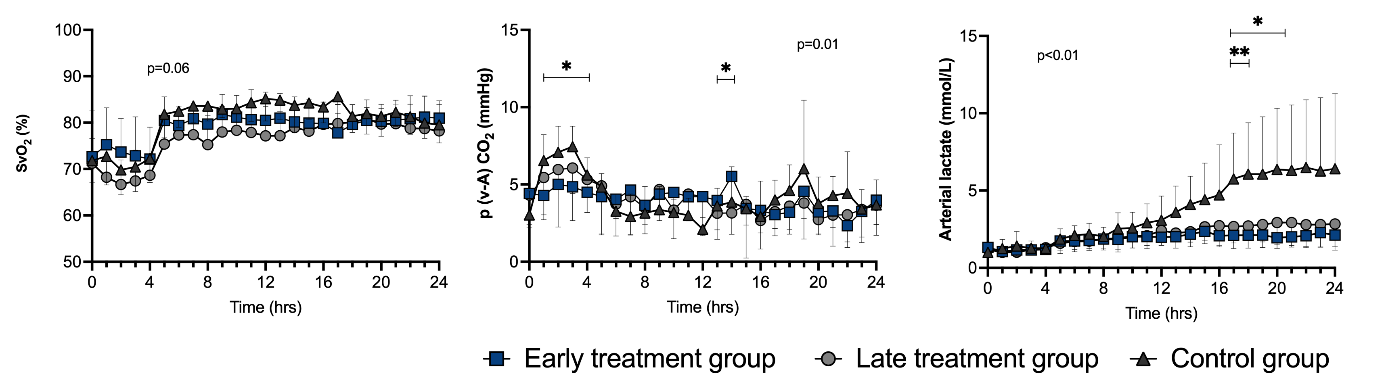


Figure S9. Platelet count (A) and kidney function (B) during the study period in the three group with the two most severe animals excluded from the control group.

Values are expressed as mean ± SD.

* p<0.05 between early-treatment and control groups in case of overall interaction

***p<0.05 between early-treatment and late-treatment groups in case of overall interaction


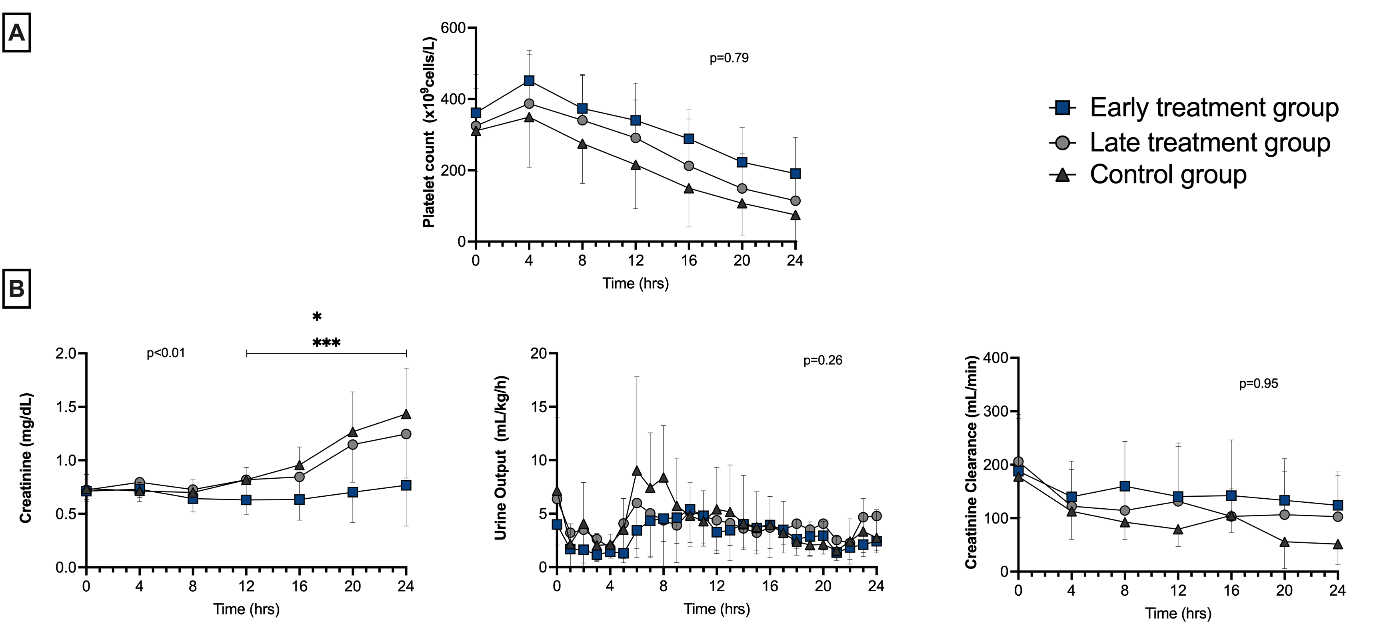


Figure S10. Changes in H3.1 nucleosome levels from baseline (ng/mL) (A) and measured cytokine levels (B) at different timepoints in the three groups with the two most severe animals excluded from the control group.

Values given are median and interquartile range; IL: interleukin

* p<0.05 between early-treatment and control groups in case of overall interaction

** p<0.05 between late-treatment and control groups in case of overall interaction


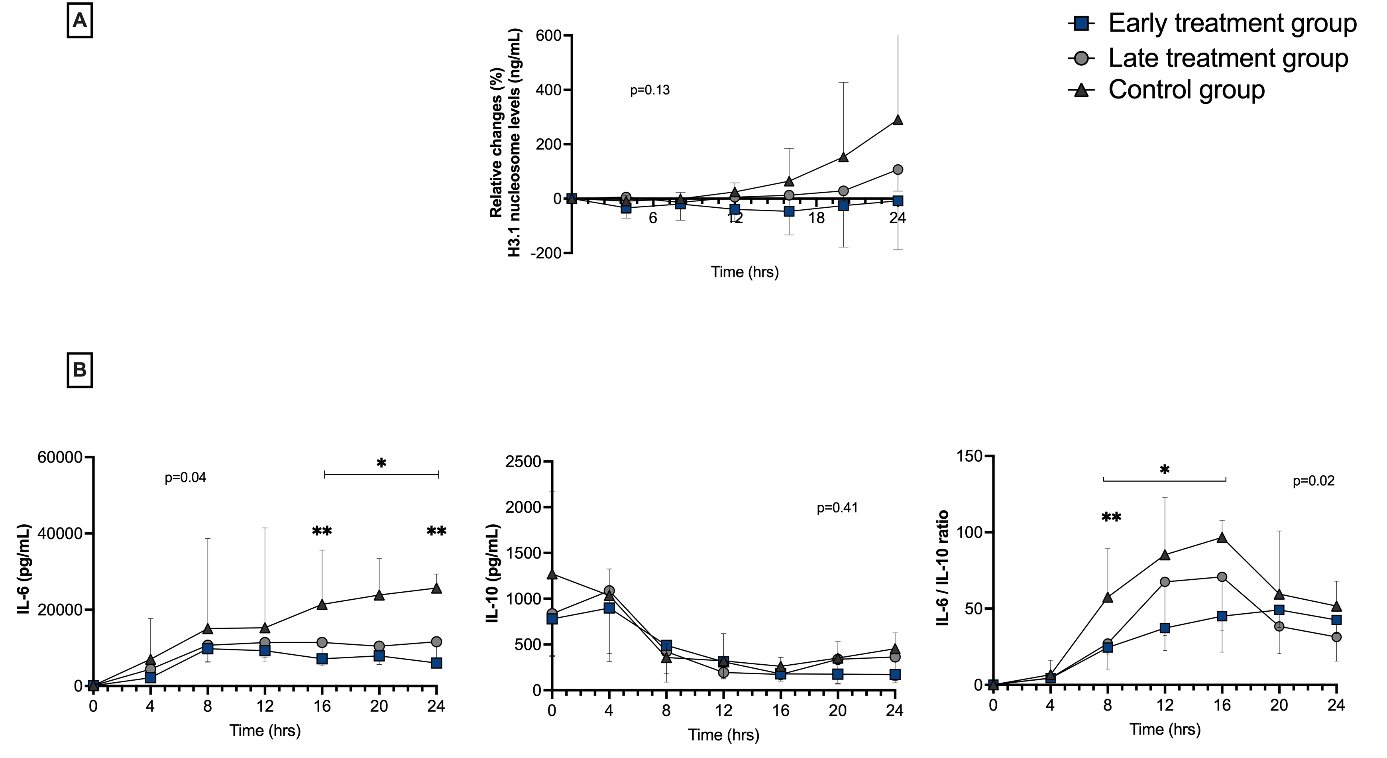


ONLINE DATA SUPPLEMENT - REFERENCES

1. He X, Su F, Taccone FS, Laporte R, Kjølbye AL, Zhang J, *et al.* A Selective V(1A) receptor agonist, selepressin, is superior to arginine vasopressin and to norepinephrine in ovine septic shock. *Crit Care Med* 2016;44:23–31.

2. Taccone FS, Su F, Deyne CD, Abdellhai A, Pierrakos C, He X, *et al.* Sepsis is associated with altered cerebral microcirculation and tissue hypoxia in experimental peritonitis. *Crit Care Med* 2014;42:e114-22.

3. Taccone FS, Su F, Pierrakos C, He X, James S, Dewitte O, *et al.* Cerebral microcirculation is impaired during sepsis: an experimental study. *Crit Care* 2010;14:R140.

4. Garcia B, Su F, Manicone F, Dewachter L, Favory R, Khaldi A, *et al.* Angiotensin 1–7 in an experimental septic shock model. *Crit Care* 2023;27:106.

5. Hong SB, Koh Y, Lee IC, Kim MJ, Kim WS, Kim DS, *et al.* Induced hypothermia as a new approach to lung rest for the acutely injured lung. *Crit Care Med* 2005;33:2049–2055.

6. Duburcq T, Durand A, Tournoys A, Gnemmi V, Gmyr V, Pattou F, *et al.* Sodium lactate improves renal microvascular thrombosis compared to sodium bicarbonate and 0.9% NaCl in a porcine model of endotoxic shock: an experimental randomized open label controlled study. *Ann Intensive Care* 2018;8:24.
